# Forearm Volume Changes Estimated by Photo-Plethysmography During an Original Candlestick/Prayer Maneuver in Patients With Suspected Thoracic Outlet Syndrome

**DOI:** 10.3389/fphys.2021.652456

**Published:** 2021-04-13

**Authors:** Jeanne Hersant, Pierre Ramondou, Victoire Chavignier, Axelle Chavanon, Mathieu Feuilloy, Jean Picquet, Samir Henni, Pierre Abraham

**Affiliations:** ^1^Vascular Medicine, University Hospital, Angers, France; ^2^UMR CNRS 1083 INSERM 6214, LUNAM University, Angers, France; ^3^School of Electronics (ESEO), Angers, France; ^4^UMR CNRS 6613 LAUM, Le Mans, France; ^5^Service of Thoracic and Vascular Surgery, University Hospital, Angers, France; ^6^Sports and Exercise Medicine, University Hospital, Angers, France

**Keywords:** plethysmography, peripheral vessel, hemodynamic response, movement, upper extremities, classification, thoracic outlet syndrome

## Abstract

**Objective:** Hemodynamic investigations in thoracic outlet syndrome (TOS) remain difficult, even in trained hands. Results are generally reported as either presence or absence of venous compression. In fact, in patients with suspected TOS but without chronic venous occlusion, the forearm volume changes may result from various combinations of forearm position from heart level, arterial inflow, and/or venous outflow positional impairment.

**Design:** Cross sectional, retrospective, single center study, accessible on Clinicaltrial.gov under reference NCT04376177.

**Material:** We used venous photo-plethysmography (V-PPG) in 151 patients with suspected TOS. The subjects elevated their arms to the “candlestick” (Ca) position for 30 s and then kept their arm elevated in front of the body for an additional 15 s (“prayer” position; Pra). This CA–Pra procedure was repeated three times by each patient with recording of both arms.

**Method:** We classified V-PPG recordings using an automatic clustering method.

**Result:** The blinded clustering classification of 893 V-PPG recordings (13 missing files) resulted in four out of seven clusters, allowing the classification of more than 99% of the available recordings. Each cluster included 65.73, 6.16, 17.13, and 10.8% of the recordings, respectively.

**Conclusion:** Venous hemodynamic profiles in TOS are not only either normal or abnormal. With V-PPG, four clusters were observed to be consistent with, and assumed to result from, the four possible associations of presence/absence of arterial inflow/venous outflow positional impairment: (1) normal response (maximal emptying in Ca and Pra), (2) isolated inflow impairment (emptying in Ca and filling in Pra due to post-ischemic vasodilation), (3) isolated venous outflow impairment (emptying then filling in Ca due to arterial inflow and emptying in Pra), and (4) simultaneous inflow/outflow impairment (emptying in Ca but no filling due to concomitant inflow impairment and further emptying in Pra).

## Introduction

Movements of the upper limb may be responsible for the compression of the neural plexus and/or vascular structures at different levels (costoclavicular, pectoris minor, humeral head, etc.). Thoracic outlet syndrome (TOS) is the symptomatic form of this positional neurovascular conflict. Its treatments are the source of the highest number of complaints against thoracic surgeons (Ferguson, 1982). Many vascular TOS are revealed during thrombotic or embolic complications (Jones et al., 2019; Ohman and Thompson, 2020), but evidence exists that uncomplicated venous TOS (Mc-Cleery syndrome) (Illig et al., 2016) can be improved with appropriate treatment (Likes et al., 2014; Moore and Wei Lum, 2015; Ryan et al., 2018; Wooster et al., 2019).

Various procedures exist to induce and standardize the positional and transient compression of neuro-vascular structures, among which the candlestick (or surrender) position is largely used. In positional analysis, venous ultrasound is difficult (Moore and Wei Lum, 2015), and dependent on the sonographer's experience. Furthermore, ultrasounds provide little evidence of forearm volume changes. Indeed, it is likely that symptoms in case of venous compression are not mainly related to the positional venous compression itself but rather to its hemodynamic consequences (i.e., forearm swelling). Thereby, measuring forearm volume changes during dynamic positional maneuvers is required. Plethysmography is a non-invasive, observer-independent tool that has been largely used in venous diseases (Winsor and Winsor, 1985). Various plethysmography techniques exist, among which photo-plethysmography (PPG) estimates the limb volume changes by measuring changes in light reflection at the skin level. Although venous PPG (V-PPG) has been used to study the hemodynamic consequence of upper limb thrombosis in Paget–Schroetter syndromes, reports of V-PPG use for positional venous investigations in non-complicated TOS are rare (Antignani et al., 1990). Since the observations of Antignani et al. (1990) the hemodynamic consequences of the positional maneuvers in patients with suspected TOS have received little attention (Illig et al., 2013; Chen et al., 2014). One reason for this is that PPG is a semi-quantitative technique, leading to question the fact that during the candlestick maneuvers the volume decrease observed with PPG corresponded to completely emptied forearm veins during arm elevation above heart level. Then, in our routine use of V-PPG in patients with suspected TOS, we used a maneuver for which, after the classical candlestick position (Ca), we asked the patients to keep their arm elevated but join them in front of their face (prayer position: Pra). We hypothesized that, in the case of venous outflow impairment (incomplete venous emptying) in Ca, Pra would result in further venous emptying and that the Ca–Pra maneuver would thereby improve V-PPG interpretation.

Our objective was to use automatic clustering to classify the V-PPG recordings observed during the Ca–Pra maneuver in patients with suspected TOS and estimate the distribution of recordings among each of the clusters observed. This classification is a first step for the objective interpretation of V-PPG results and is essential for the future potential use of V-PPG as a diagnostic test in patients suspected of TOS.

## Methods

We retrospectively analyzed the data files of a cross-sectional study of all patients who were referred to our laboratory for vascular investigation (University Hospital in Angers) from January 1 to December 31, 2019, with suspected TOS. Indeed what we aimed was to test the effects of positional changes on non-chronically occluded vessels and not the consequences of vessel chronic occlusions.

### Ethical Standards

This study was performed in compliance with the principles outlined in the Declaration of Helsinki and validated by the Ethics Committee in Angers (reference: 2020/17 and accessible on Clinicaltrial.gov under reference NCT04376177). As an observation of our medical routine and in accordance with French law, no individual consent was required, but all the patients were fully informed that they could deny the use of their medical file for research purposes. Patients denying the use of their data, unable to understand the information for linguistic or cognitive reasons, and under 18 years of age were not included in the analysis.

### Experiment Design

We recorded patient demographics and health conditions including age, sex, weight, and height. History of chest, shoulder, or arm trauma or surgery and any ongoing treatments were also included. The patients self-completed the “disability of the arm and shoulder” (DASH) 38-item questionnaire.

**Figure 1:**
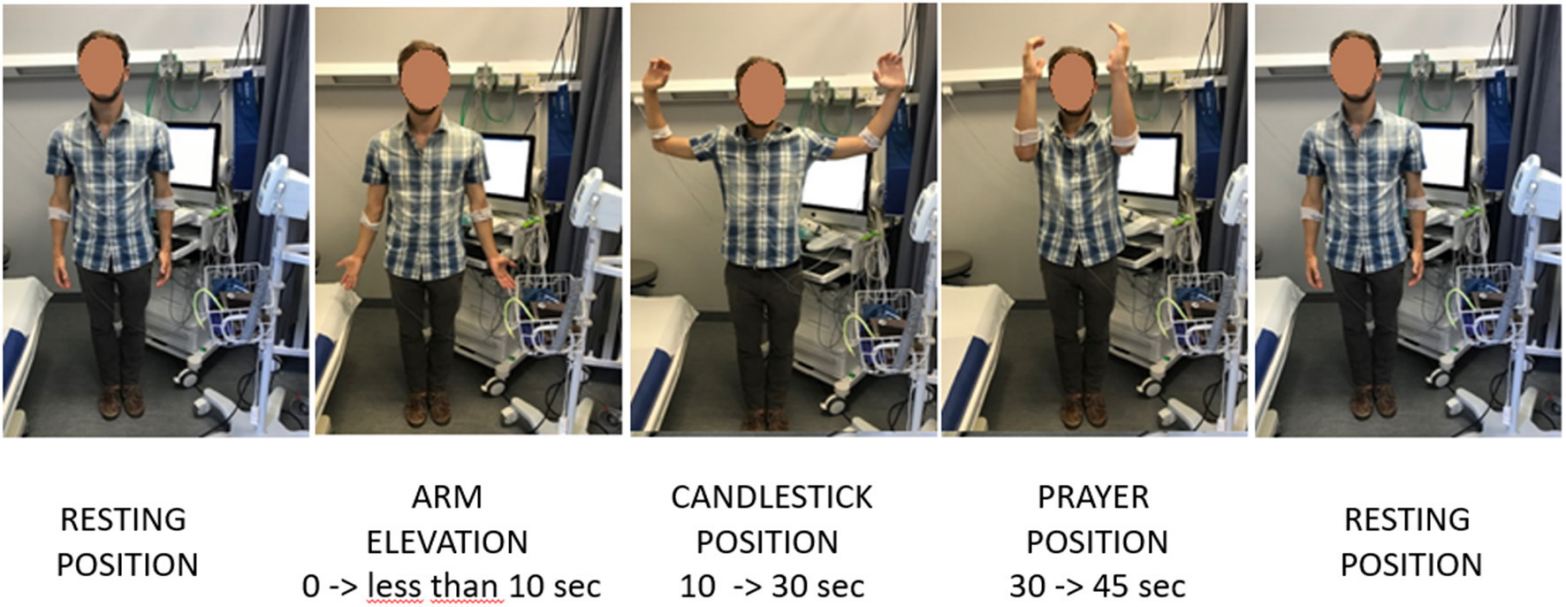
Procedures of arm positioning used in the present study.

The provocative maneuvers consisted of four consecutive, distinct periods. After standing still in the resting position, the patients were required to raise their arms slowly to 90° of abduction in <10 s, with the arms fully externally rotated and the elbows at 90° of flexion and flexed slightly behind the frontal plane. We primarily used this candlestick maneuver since it seems to exhibit both good sensitivity and good reliability in clinical practice. We assumed that, in the absence of venous outflow impairment, this would result in emptying the forearm venous system. The patients maintained this “Ca” position until the 30th second (second period). Then, we asked them to move their elbow rapidly in front of them without lowering the arms. This “prayer” (Pra) position aimed to keep the arm above heart level while opening the costo-clavicular angle, thereby emptying the veins if venous outflow impairment occurred in the Ca position (third period). At the 45-s mark, the arms were lowered to their initial position (fourth period). Figure 1 illustrates the procedure of arm movements used in the study. Each subject performed the complete procedure three times with a 2-min resting period between each test to confirm the individual reliability of recordings.

### V-PPG Recordings

V-PPG recordings (4 Hz sampling rate) on each forearm were performed with Vasolab320 (ELCAT, Germany). The system, once stabilized, starts from zero, records V-PPG values for 60 s, and stops automatically. The manufacturer's program is designed to be used in lower limb investigations of venous reflux, and recording duration cannot be increased, while simultaneous recording of arterial pulsatility with digital arterial PPG is impossible. Increases from zero denote forearm emptying and vice versa. Values are expressed in arbitrary units (AU).

### Data Extraction and Normalization

All V-PPG files recorded in 2019 were exported as anonymous text files for analysis. Since the recording of arm lowering was too short to allow the return toward baseline value and presented occasional transient peaks likely due to movement artifacts, data normalization was performed using the maximal value observed on each arm in the first 45 s of the procedure as 100%, and clustering analysis was done after exclusion of the arm lowering (4th) period.

### Analysis of Data and Clustering of PPG Patterns

Recordings of all the arms were analyzed statistically using clustering, a technique commonly used in data time series analysis. Also called “unsupervised classification,” clustering can be defined as the organization of patterns into coherent groups. Clustering automatically discovers and identifies groups (called clusters) in a data set such that the data in each cluster are similar, and dissimilar data belong to different clusters. A detailed presentation is available open-access (Bruneau et al., 2013).

In order to reduce the sensitivity of partitioning to noise, each waveform was denoised by a moving average with a window size of 2.5 s (10 samples). As a first step of the clustering analysis, for each of the three first periods of the Ca–Pra maneuver, the slope of the waveform was used to characterize each period by a discretized symbol. Thus, each record was defined by three symbols, and the first partitioning step considered each group as the waveforms with the same slope values (integers). This partitioning allowed the initial number of records to be reduced to a limited number of types. Within each type, a waveform was obtained by averaging all records belonging to each type. The next step consisted of submitting the various types to hierarchical agglomerative clustering in order to merge them into classes. This approach determines successive levels of classes from an initial set of pre-established types until obtaining a single class. At each level, it organizes the classes by merging the two most similar profiles. This approach organizes the classes into a hierarchical structure according to the similarity measures based on correlation distance. These classes were obtained by considering the optimal partition that maximizes and minimizes, respectively, the between-class and within-class variances. Within each class, a waveform was obtained by averaging all types belonging to each class.

The last step submitted the classes to the same clustering algorithm in order to focus on the final clusters that optimized the same criterion. As done previously, within each final cluster, a curve was obtained by averaging all recorded waveforms belonging to this cluster.

Once the clusters were defined, we evaluated the partition obtained with the Silhouette coefficient. The Silhouette coefficient can be used to analyze the relevance of the membership of the records to final clusters. For each record, this coefficient (Si) measures the similarity between this record and other records in its own cluster when compared to records from other clusters:

$$\begin{array}{l} S_{i}=\;\frac{b_{i}-a_{i}}{\max\left\{a_{i},b_{i}\right\}} \end{array}$$

with *a*_*i*_ being the average distance from the *i*^*th*^ record to the other records in the same cluster as *i*^*th*^record and *b*_*i*_ being the minimum average distance from the *i*^*th*^ point to points in the closest different cluster. The *S*_*i*_ coefficient ranges between [−1] and [1], where 1 indicates an appropriate assignment to cluster and −1 indicates a misclassification. The Silhouette coefficient was computed for all records. The clustering analysis was performed by one investigator (MF) blinded from the clinical results and expected patterns.

The data are presented as numbers (percentages), medians (25 and 75 percentiles), or means ± standard deviations. A comparison of between-arm differences of absolute V-PPG values was done with Bland Altman representation. All statistical analyses were performed using SPSS (IBM SPSS Statistics, V15.0, Chicago, IL, United States) and Matlab R2019A (Matworks Inc., United States). For all tests, a two-tailed *P* < 0.05 was considered to be statistically significant.

## Results

Among the eligible patients (*n* = 157), six did not have V-PPG recordings for technical reasons or denied the use of their medical file and therefore were excluded from the study. The remaining 151 patients were 38.8 ± 12.5 years old and predominantly female (*n* = 108). Sixty-nine patients reported bilateral pain by history. A description of the population is reported in Table 1. As shown, 69 patients (45.7%) reported a history of chest, shoulder, or arm trauma or surgery. Many differences were observed between males and females. Specifically, while all Doppler recordings confirmed the presence of arterial or venous compression in males, approximately only half of the results were found positive in females.

**Table 1:** Characteristics of the population and comparison by gender.

	**Females**	**Males**	**Population**	***P* value**
	***n* = 108**	***n* = 43**	***n* = 151**	
Age (years old)	37.1 ± 12.2	43.2 ± 12.4	38.9 ± 12.5	0.007
Right hander	95 (88)	41 (95.4)	136 (90.1)	0.171
Height (cm)	164 ± 10	177 ± 100	167 ± 11	0.001
Weight (kg)	61.9 ± 21.2	64.0 ± 23.5	62.5 ± 21.8	0.589
Right systolic blood pressure (mmHg)	125 ± 15	128 ± 13	126 ± 14	0.335
Right diastolic blood pressure (mmHg)	73 ± 17	73 ± 24	73 ± 19	0.948
Left systolic blood pressure (mmHg)	122 ± 23	130 ± 13	124 ± 21	0.128
Left diastolic blood pressure (mmHg)	71 ± 24	71 ± 24	71 ± 24	0.983
Active smoker	38 (35.2)	26 (60.5)	64 (42.4)	0.002
History of chest, shoulder, or arm trauma or surgery	46 (42.6)	23 (53.5)	69 (45.7)	0.225
Pain killers on a regular basis	54 (50)	19 (44.2)	73 (48.3)	0.519
DASH score	92 ± 43	81 ± 43	89 ± 43	0.008
Already had physiotherapy at the date of referral	85 (78.7)	26 (60.5)	111 (73.5)	0.022
Normal Doppler	51 (47.2)	0 (0.0)	51 (33.8)	0.001
Doppler positive for arterial compression on the right side	37 (34.3)	25 (58.1)	62 (41.1)	0.007
Doppler positive for arterial compression on the left side	39 (36.1)	23 (53.5)	62 (41.1)	0.051
Doppler positive for venous compression on the right side	3 (2.8)	33 (76.7)	36 (23.8)	0.001
Doppler positive for venous compression on the left side	0 (0.0)	35 (81.4)	35 (23.2)	0.001
Pain on the right side	43 (39.8)	20 (46.5)	63 (41.7)	0.451
Pain on the left side	46 (42.6)	21 (48.8)	67 (44.4)	0.485
Fatigability on the right side	28 (25.9)	21 (48.8)	49 (32.5)	0.007
Fatigability on the left side	33 (30.6)	24 (55.8)	57 (37.7)	0.004
Weakness/heaviness on the right side	30 (27.8)	12 (27.9)	42 (27.8)	0.987
Weakness/heaviness on the left side	27 (25.0)	14 (32.6)	41 (27.2)	0.346
Paresthesia on the right side	36 (33.3)	18 (41.9)	54 (35.8)	0.324
Paresthesia on the left side	39 (36.1)	20 (46.5)	59 (39.1)	0.237
Peak PPG on the right side (arbitrary units, AU)	13.1 ± 8.3	15.1 ± 11.1	13.7 ± 9.2	0.010
Peak PPG on the left side (AU)	13.1 ± 8.1	14.2 ± 10.6	13.4 ± 9.0	0.001

*Results are mean ± standard deviation or number (percentages)*.

**Figure 2:**
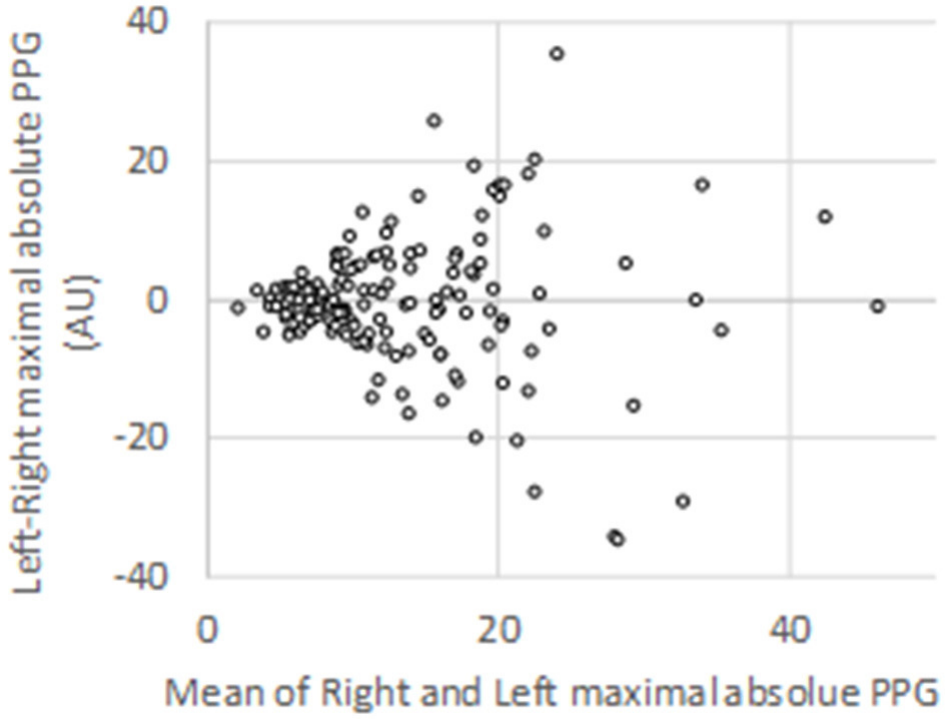
Bland–Altman representation of the absolute peak values observed on each arm for the 151 patients studied during photo-plethysmography recordings.

Among the 906 expected V-PPG recordings (three recordings per patients on both sides), 13 were missing due to probe disconnection, corrupted electronic file, or impossibility of performing all three usual procedures due to pain. This resulted in 893 files for the clustering analysis supplied by 151 patients. On average, there was no difference between the maximal absolute PPG values observed on the right (13.7 ± 9.2 AU) and left (13.4 ± 8.9 AU) arm before normalization. Nevertheless, inter-individual variability (with values ranging from 1.5 to 28.4 AU) as well as between-arm variability (standard deviation to the mean of between-arm difference = 9.7 AU) was wide (shown in Figure 2).

**Figure 3:**
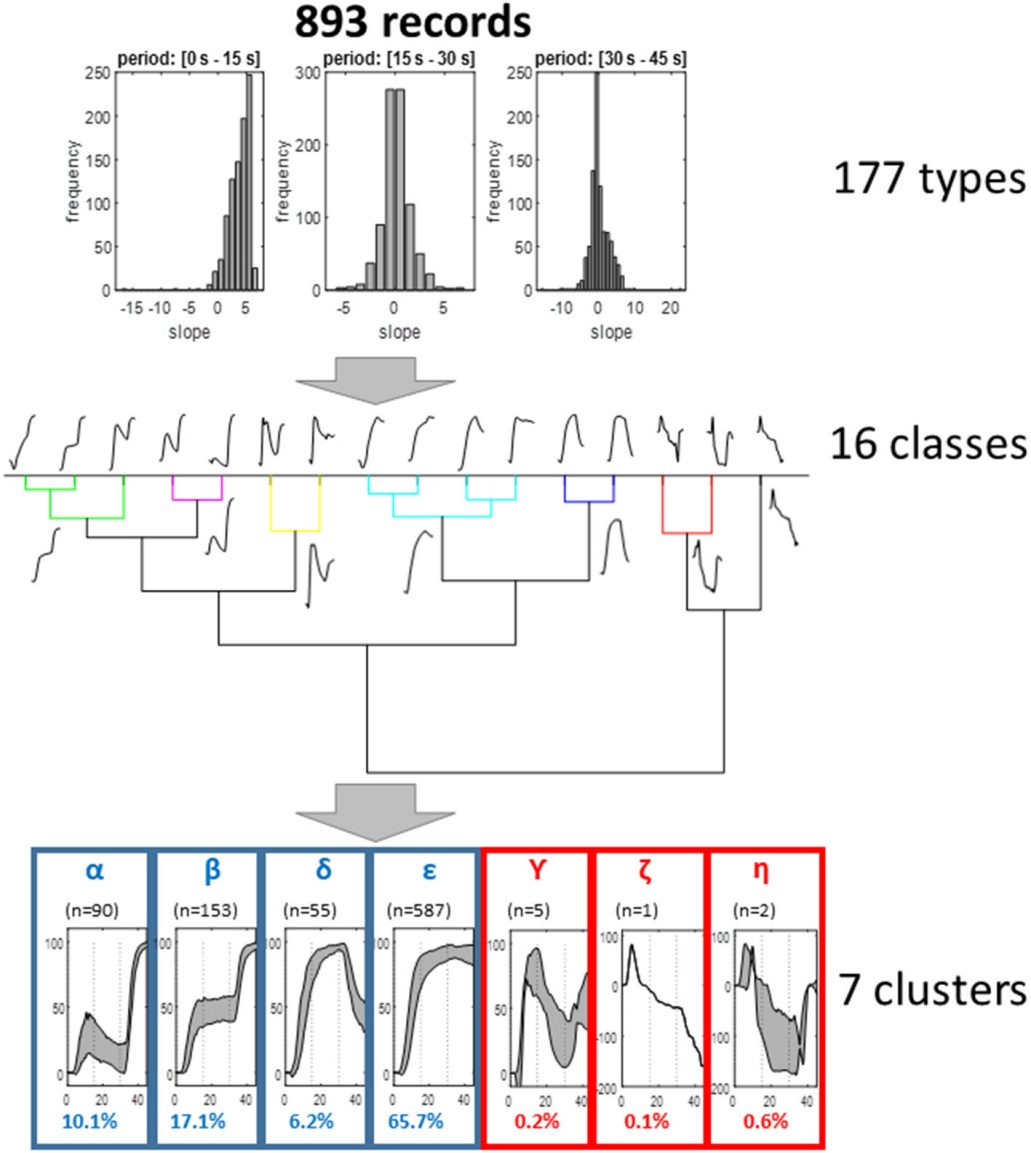
Representation of the different steps of the clustering analysis. As shown, the analysis resulted in seven clusters, four of which allowed the classification of more than 99% of recordings and appeared of clinical interest. For these four clusters, the representation is the interval 25° and 75° centile of the different values (gray zone); the other three are mean values.

As shown in Figure 3, the first step of the analyses reduced the 893 recording to 177 groups. The second step resulted in the finding of 16 different classes. The last step resulted in the observation of seven clusters. Three of these clusters (γ, η, and ζ) included <10 of the recordings. Then the four patterns (α, β, δ, and ε) made possible the classification of 99.1% of the total recordings.

The α, β, δ, and ε clusters included 10.08, 17.13, 6.16, and 65.73% of the initial available recordings, respectively.

**Figure 4:**
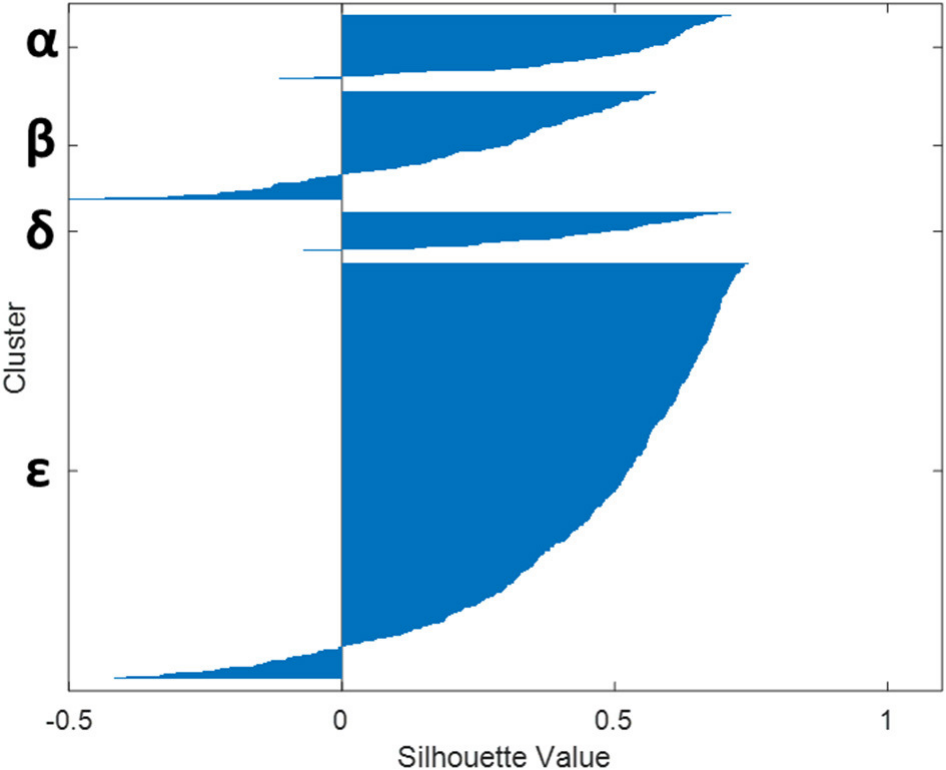
Relevance of the four clusters (α, β, δ, and ε) obtained as assessed by the observation of the Silhouette coefficient on the records.

Lastly, Figure 4 presents the Silhouette coefficient and shows homogenous clusters, where there are only 10% of records that could possibly be in other clusters (other than the one where it finally belongs).

## Discussion

This is the first study classifying usual V-PPG changes observed during a specific Ca–Pra procedure derived from the candlestick maneuver in patients with suspected TOS. Automatic classification of V-PPG patterns observed during the Ca–Pra maneuver resulted in four different clusters.

Plethysmography is a generic term for the techniques measuring limb volume changes under various stimulating conditions (Berthelot et al., 1885–1902; Winsor and Winsor, 1985). PPG exists since the 1930s (Hertzman and Spealman, 1937) and uses a light source that is reflected as a function of the skin blood volume. The technique is easy and of low cost, although it produces semi-quantitative estimation of the limb volume with the amplitude of the changes observed being of low reliability in test–retest recordings. There are many reports of A-PPG detecting the changes in pulse amplitude at the finger level in normal and TOS-suspected patients (Colon and Westdorp, 1988). With low-pass filters, slow but ample changes associated to venous filling/emptying (V-PPG) become measurable. There have been some evaluations of V-PPG in upper limb thrombosis (Sullivan et al., 1983; Mukherjee et al., 1991), but, to the best of our knowledge, there is only one published case series of V-PPG in TOS in which limitations have been previously underlined (Antignani et al., 1990). The specific interest here is that we studied only the pattern of V-PPG changes, regardless of the amplitude of these changes, during a slightly modified candlestick maneuver to improve PPG results.

**Figure 5:**
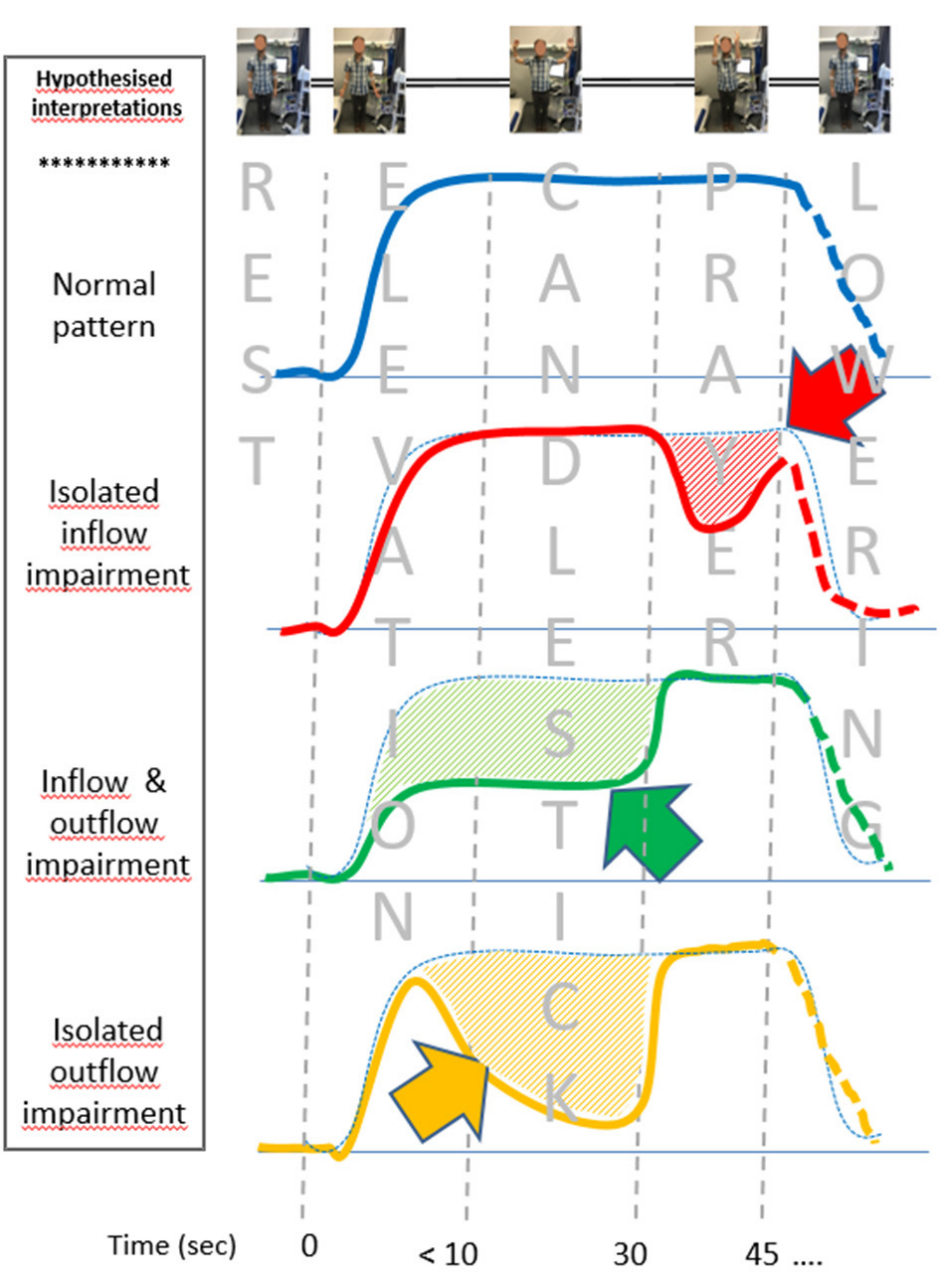
Schematic representations of the venous photo-plethysmography pattern interpretation, depending on the presence of inflow and/or outflow impairment (emptying is an upward change, while filling is a downward change). Differences from the assumed normal pattern (blue dot points) are underlined by hash bars and arrows. Isolated inflow impairment during the candlestick phase is assumed to result in post-ischemic vasodilation. The green figure is expected to result from arrested inflow and outflow during the candlestick that are normalized during the prayer position. Outflow impairment with persistent inflow results in forearm filling, followed by emptying at the prayer position (yellow figure). The lowering was not standardized and might be different in each pattern and thus presented as dash lines.

From a physio-pathological point of view, while we acknowledge that the V-PPG signal is sensitive to skin changes only and can vary with tissue edema, the present study considered V-PPG changes as changes in venous volume of the limb. We believe that the observation of four clusters is not surprising. Indeed our assumption is that, in the absence of arterial or venous inflow/outflow impairment, the veins would empty with arm elevation above heart level (both in the Ca and Pra positions) and would remain emptied until the arm was lowered, as observed in the type “ε” cluster. Then, we assume that the “ε” cluster is showing normal hemodynamic results. It should be kept in mind that this normal hemodynamic response does not exclude the possibility that a venous or arteriovenous positional compression occurred during the candlestick position. Indeed collateral vessels bypassing the level of venous or arteriovenous positional occlusion or the presence of a pre-clavicular cephalic vein or of a vein emptying in the external jugular vein may normalize venous outflow. The former is frequent in chronic occlusion of the veins but could be observed as an adaptive phenomenon in the case of iterative, transient positional venous compression. The latter has been reported to occur in between 1 and 10% of the general population (Anastasopoulos et al., 2015; Steckiewicz et al., 2015; Shetty et al., 2016; Novakov and Krasteva, 2018). The δ cluster corresponds to forearm volume increase during the “prayer” phase of the maneuver. The most intuitive explanation for this is that arterial inflow impairment occurred in the candlestick position, while venous outflow was preserved (red line in Figure 5). We believe that this volume increase during the prayer position results from post-ischemic vasodilation. We recognize that we cannot exclude the possibility that the δ pattern could also result from isolated venous outflow impairment, occurring only in the prayer position. Simultaneous recording of A-PPG and V-PPG is needed to confirm our hypothesis, but this is impossible with our system.

We assume that both the β and α clusters result from venous outflow impairment in the candlestick position. Indeed, in both cases, moving the elbows forward resulted in further emptying of the forearm veins, suggesting incomplete emptying during the Ca position. The difference between the α cluster and β cluster is observed on the “candlestick” phase where the forearm volume remains stable in the β cluster (green line of Figure 5), while the arm slowly fills (volume increases) in the α cluster, as shown with the orange line of Figure 5. We advocate that this implies that, in the β cluster, both arterial inflow and venous outflow were impaired, while in the α cluster, the artery remained patent and fills the veins until venous outflow was restored when the arms were moved to the “prayer” position. Once again, a simultaneous recording of arterial and venous PPG would confirm this assumption, and future studies are needed with devices that allow such simultaneous analyses.

There are the limitations to the present study. First, PPG is a semi-quantitative surface technique that can be influenced by skin pigmentation or probe position, while other plethysmography techniques are able to provide more quantitative measurements. Nevertheless, if dealing with the pattern of volume changes regardless of absolute values, we believe that PPG provides an easy and low-cost approach. Furthermore, all plethysmography techniques cannot be easily applied with the arm in movement.

Second, the results observed here are specific for the use of the Ca–Pra maneuver and not applicable to another series of movements. Evaluation of V-PPG results during other maneuvers is required. The specific interest of the present Ca–Pra modified candlestick test is the Pra phase that allows confirmation of venous emptying during the Ca phase and seems to discriminate arterial from venous inflow/outflow hemodynamic results.

Third, we provide no evidence of the clinical benefit of this technique to date. We advocate that the present retrospective analysis was required before proposing a prospective study integrating results classified according to the present clustering. The automatic classification of the patterns observed during routine recordings would facilitate clinical practice and allow for some prospective studies analyzing the interest of these different hemodynamic profiles on diagnosis, the treatment choices, and treatment evaluations of patients with TOS.

Fourth, we provide no comparison with symptoms or other investigations. Comparison with symptoms is not optimal because it is likely that many symptoms did not result from TOS and that, on the contrary, dominant symptoms on one side may mask contralateral symptoms in patients, with PPG results suggesting bilateral flow impairment. Comparison with other investigations is also debatable. Indeed it is important to keep in mind that V-PPG provides only hemodynamic results and allows neither the visualization of the level of the compression nor the detection of positional incomplete compression or positional complete occlusions but with collateral pathways. Contrast-injected radiology and ultrasound are necessary to visualize the compression level. Nevertheless, on one hand, ultrasound can be challenging, even in thrombotic complications resulting from TOS (Moore and Wei Lum, 2015), and does not correlate nor measure forearm volume (Czihal et al., 2015). On the other hand, angiography is only a pre-surgical approach; it cannot be proposed as a systematic tool in patients with suspected TOS.

Fifth, the distribution of the recordings among the observed clusters does not necessarily represent the proportions that would be found in the general population nor the one that would be observed in patients with proven TOS. Similarly, we did not compare our patients with normal subjects with suspected TOS. Indeed the presence of a vascular compression in the general population and in apparently asymptomatic subjects is high, and including control subjects would not exclude the possibility that some would show positive results. Furthermore, we aimed at having a significant number of positive (abnormal) tests and focusing on the population that might benefit PPG investigation.

Sixth and unexpectedly, we observed some clinical differences between the males and the females included in the present study, specifically in the prevalence of positive results in the two sub-groups. We have no obvious explanation for these differences, and it is very likely that the males and females would have different proportions in the distribution of recordings among the observed clusters. There is also a possibility that the clustering might result in different average patterns between males and females. We do not believe that it is the case because, to the best of our knowledge, no study ever reported differences in diagnostic criteria of TOS between males and females. Unfortunately, the number of males is relatively small in the present study for a valid clustering classification by gender. As a result, future studies are required to confirm whether or not clustering and the resulting average patterns might differ between males and females.

Seventh and lastly, from a mathematical point of view, it is possible that other statistical approaches of data mining would result in different classifications, but the clustering approach is largely used to define classes in time series. The interest of the clustering approach is to provide an observer-independent classification, and the Silhouette allows for a verification of the robustness of the findings. The classification observed needs to be externally confirmed with another population and tested with another method.

Despite these limitations, there are important potential applications of the present study. On one hand, TOS remains difficult to diagnose, and its diagnosis relies on a holistic approach in which any additional information can help to better argue in favor of movement-induced hemodynamic changes being the cause of symptoms in the patient. The interest of V-PPG during the Ca–Pra maneuver, among the other tests that participate to the holistic diagnostic approach or TOS, requires future analyses. On the other hand, surgery of TOS is the operation that is the root of the highest number of patient complaints against their surgeons (Ferguson, 1982). Objective recording of hemodynamic results could be not only of clinical interest but also of legal interest in case of a complaint to assess observer-independent evidence of the hemodynamic impairments.

In conclusion, V-PPG—a very easy technique—could be of renewed interest in the evaluation of patients suspected of having TOS with an original Ca–Pra maneuver. The Pra position unmasked incomplete emptying of forearm at the Ca position in ~25% of our observations. Whether or not a comparable proportion would be found in apparently healthy subjects is an important point to be determined by future investigations. We believe that the observation of four clusters during this specific maneuver opens original physiological hypotheses about inflow/outflow impairments in patients suspected of having TOS. Comparison of V-PPG results to simultaneous arterial recordings could be an interesting step to prove the assumptions about arterial inflow impairment that we propose as possible explanations of the results of the present study. Another next important step is to estimate the additional value of the present V-PPG classification, to clinical complaints, and other objective non-invasive measures (ultrasounds) in the diagnosis of patients with suspected TOS. Lastly, evolution of individual V-PPG profiles after (conservative or surgical) treatment remains to be done.

## Data Availability Statement

The raw data supporting the conclusions of this article will be made available by the authors, without undue reservation.

## Ethics Statement

The studies involving human participants were reviewed and approved by Ethics Committee in Angers: reference: 2020/17. The patients/participants provided their written informed consent to participate in this study.

## Author Contributions

PA and SH contributed to conceptualization. PA, MF, and SH contributed to methodology. AC, PR, JH, VC, and PA contributed to formal analysis and investigation. PA, MF, and JP contributed to writing – original draft preparation. AC, PR, JH, VC, and SH contributed to writing – review and editing. PA and MF contributed to funding acquisition. All the authors read and approved the final manuscript.

## Conflict of Interest

The authors declare that the research was conducted in the absence of any commercial or financial relationships that could be construed as a potential conflict of interest.

## Abbreviations

A-PPG: arterial volume pulsatility plethysmography
AU: arbitrary units
Ca: “candlestick” position
DASH: disability of the arm and shoulder
PPG: photo-plethysmography
Pra: “prayer” position
TOS: thoracic outlet syndrome
V-PPG: venous photo-plethysmography.
